# Post-transplant lymphoproliferative disorder presenting with multifocal aggressive osseous lesions

**DOI:** 10.1259/bjrcr.20180045

**Published:** 2018-10-02

**Authors:** James Anthony Korf, Mary-Kristen Jesse, Theodore C. Schultheiss

**Affiliations:** 1 Department of Radiology, University of Colorado at Denver–Anschutz Medical Campus, Denver, CO, USA; 2 Department of Pathology, University of Colorado School of Medicine, Denver, CO, USA

## Abstract

Post-transplantation lymphoproliferative disorder (PTLD) encompasses a broad category of lymphoid and plasmacytic proliferations that occur following solid organ and/or allogeneic stem cell transplantation. PTLD manifests in the setting of chronic immunosuppression and is thought to be associated with the Epstein Barr Virus, although Epstein Barr Virus infection or reactivation is not required for the process to occur. Pathologic correlation is necessary for diagnosis with B-cell lymphocytes the most commonly isolated cellular etiology. There is a broad range of clinical and imaging presentations of PTLD with intestinal and nodal involvement being the most common. Imaging plays an integral part in the diagnosis and management of PTLD, as it is utilized in the initial diagnosis and staging, guiding biopsy of lesions, and gauging treatment response. Presenting symptoms of PTLD are often vague and nonspecific and depend on the organ systems affected. Musculoskeletal involvement is especially rare, with only a few cases described in the literature. We present a case with multifocal osseous manifestations of PTLD occurring years after a renal living donor transplant.

## Case report

The patient is a 43-year-old female who developed end stage renal disease following an ANCA positive rapid progressive glomerulonephritis, for which she received a living donor renal transplant six years ago. The donor tissue was negative for Epstein Barr Virus (EBV serologies, while the recipient was positive for EBV IgG Antibodies only. She was managed on chronic immunosuppression with a combination of azathioprine and prednisone.

**Figure 1.**
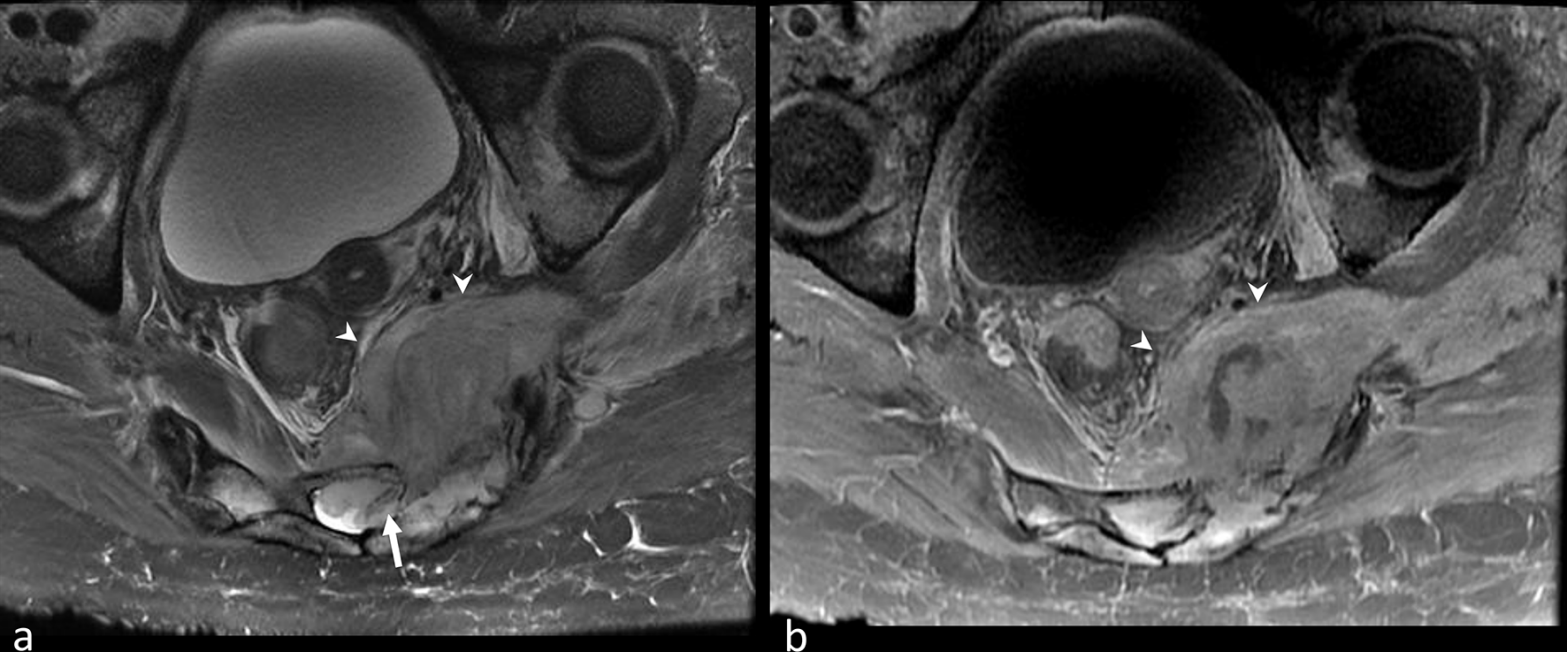
Axial *T*_2_ (a) and *T*_1_ postcontrast (b) MRI images demonstrating a *T*_2_ intermediate signal enhancing mass arising from the left sacral ala with large extra osseous soft tissue component extending into the pelvic cavity (arrowheads) and spinal canal (arrow).

**Figure 2.**
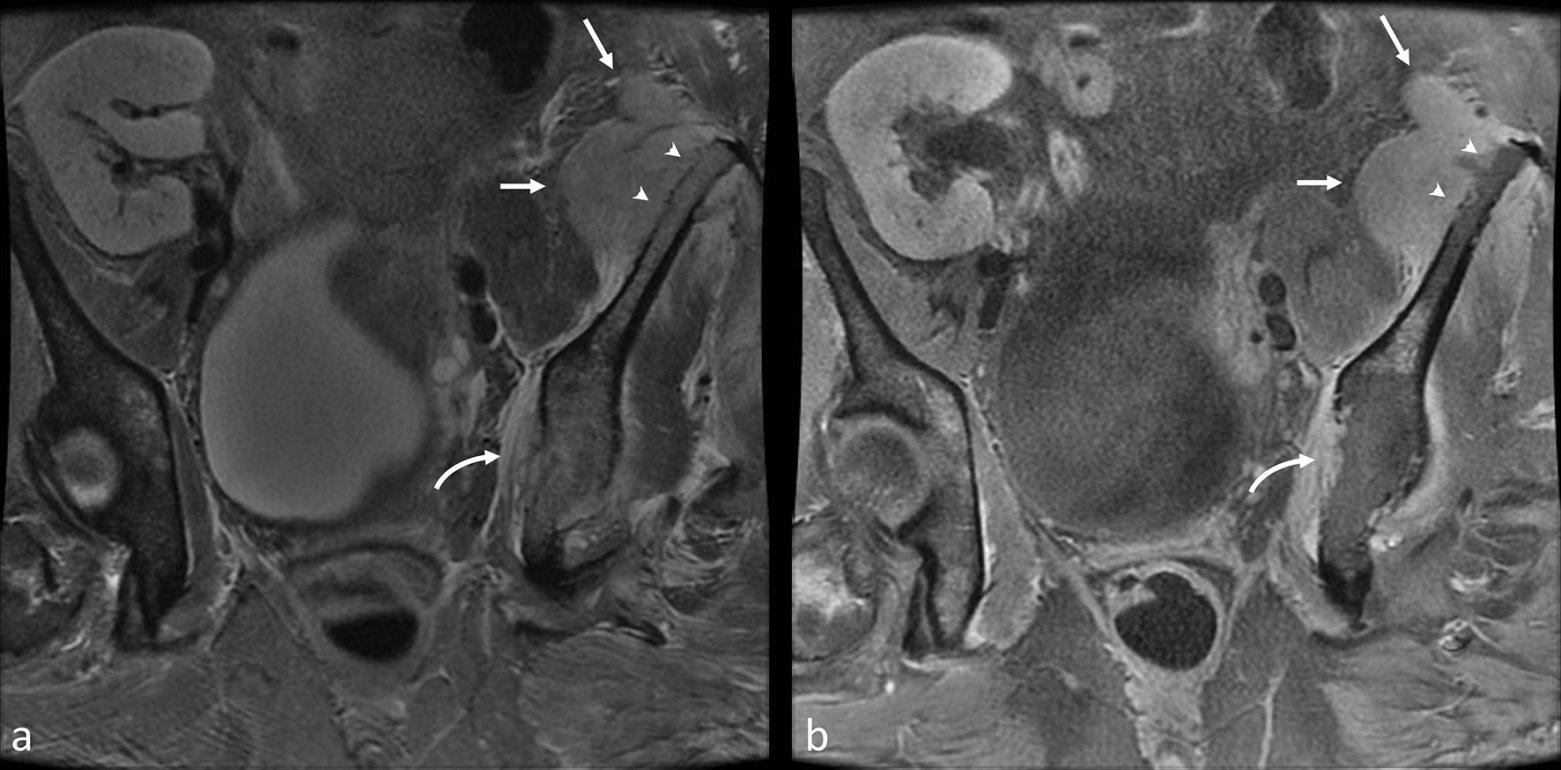
Coronal *T*_2_ (a) and *T*_1_ postcontrast (b) images demonstrate an intermediate *T*_2_ hyperintense enhancing lesion arising from the left iliac crest (straight arrows) with aggressive underlying cortical destruction (arrowheads). Similar aggressive lesion involving the ipsilateral medial acetabular wall (curved arrow).

The patient presented initially after an exacerbation of chronic lower back pain following a non-traumatic injury while dancing. Radiographs and MRI of the lumbar spine were performed as part of the initial work-up. Radiographs were negative for acute injury. MRI of her L-spine incompletely captured irregular marrow signal at her left ischium and right sacral ala. This raised the concern for a primary or metastatic marrow replacing process and prompted further imaging work-up. MRI of the pelvis and sacrum performed at this time demonstrated multiple *T*_2_-hyperintense and enhancing osseous lesions involving the lumbar spine, sacrum, pelvis, and proximal left femur. Several lesions demonstrated aggressive features, including periosteal reaction and large extra osseous soft tissue components (Figures 1 and 2). The multifocal nature of the bone involvement and the presence of lytic and aggressive features raised concern for osseous metastatic disease from a distant primary.

**Figure 3.**
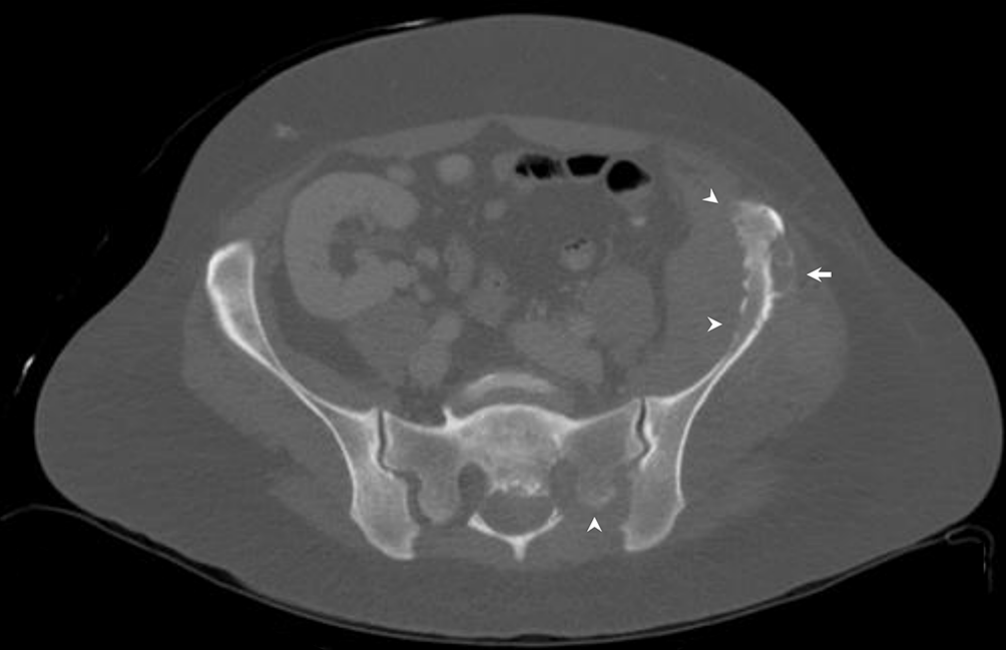
Axial CT image demonstrating the aggressive cortical destruction (arrowheads) and periosteal reaction (arrow) of the left iliac crest and destruction of the posterior left sacral ala.

**Figure 4.**
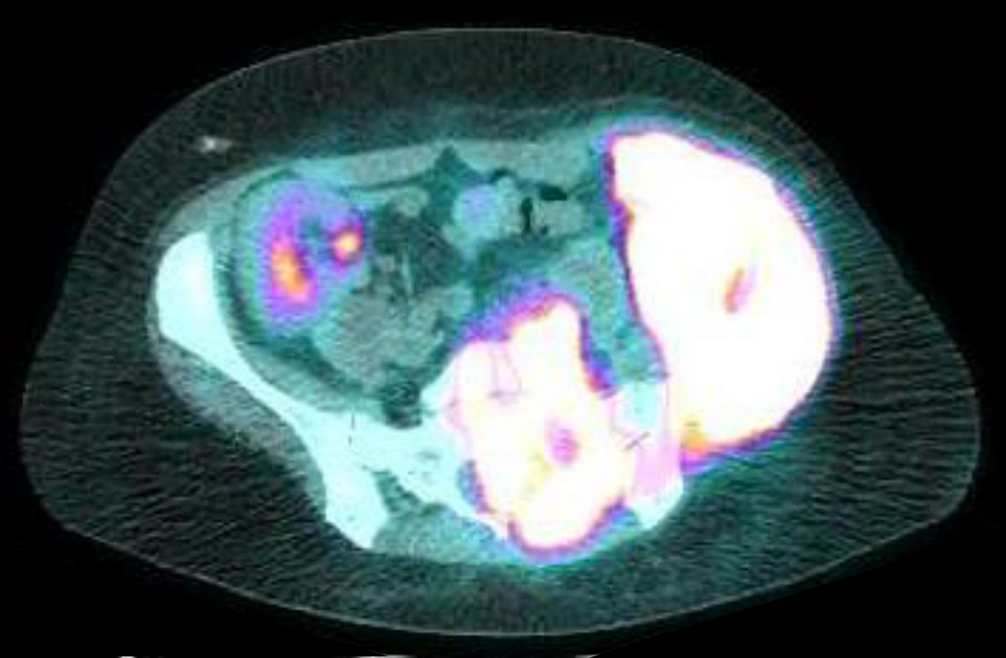
PET/CT with markedly FDG avid left iliac crest and sacral masses. FDG, fludeoxyglucose; PET, positron emission tomography.

**Figure 5.**
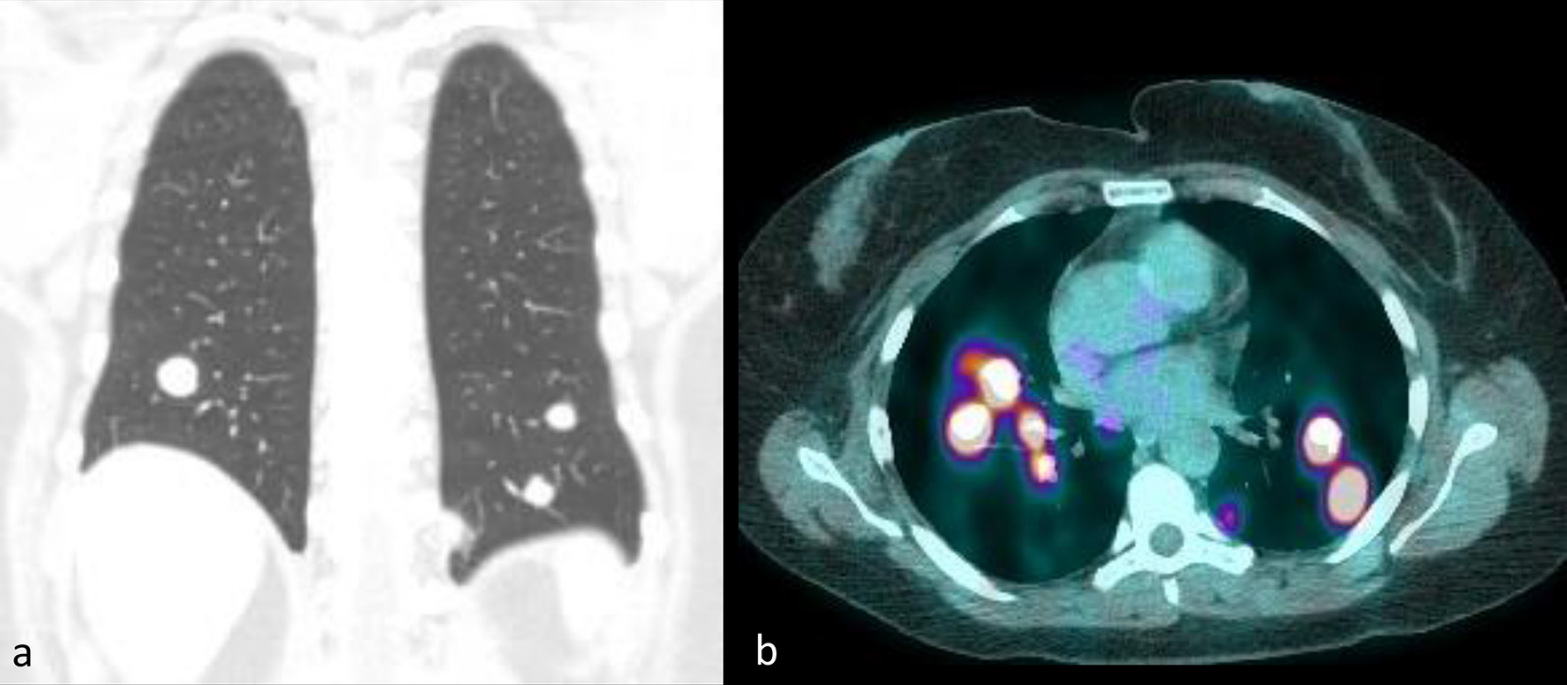
Coronal reformatted CT Chest (a) and fused FDG-PET CT (b) demonstrating the multiple bilateral round and FDG avid pulmonary nodules.

CT of the chest, abdomen, and pelvis was performed at this time in attempt to locate a primary site of disease. The CT scan again demonstrated multiple bone lesions with areas of marked cortical destruction (Figure 3). Numerous round pulmonary nodules measuring up to 1.8 cm, as well as a few enlarged axillary lymph nodes, the largest measuring 2.1 cm in the short axis, were also noted. However, there was no conclusive primary site of malignancy. Fluo-D-glucose positron emission tomography revealed markedly fludeoxyglucose (FDG) avid bone lesions with maximum standardized uptake value up to 35.8 (Figure 4). Additionally, the rounded pulmonary nodules and prominent thoracic lymph nodes were found to be FDG avid as well, with the SUVmax of the nodules and lymph nodes measuring 23.6 and 30.9, respectively (Figure 5).

**Figure 6.**
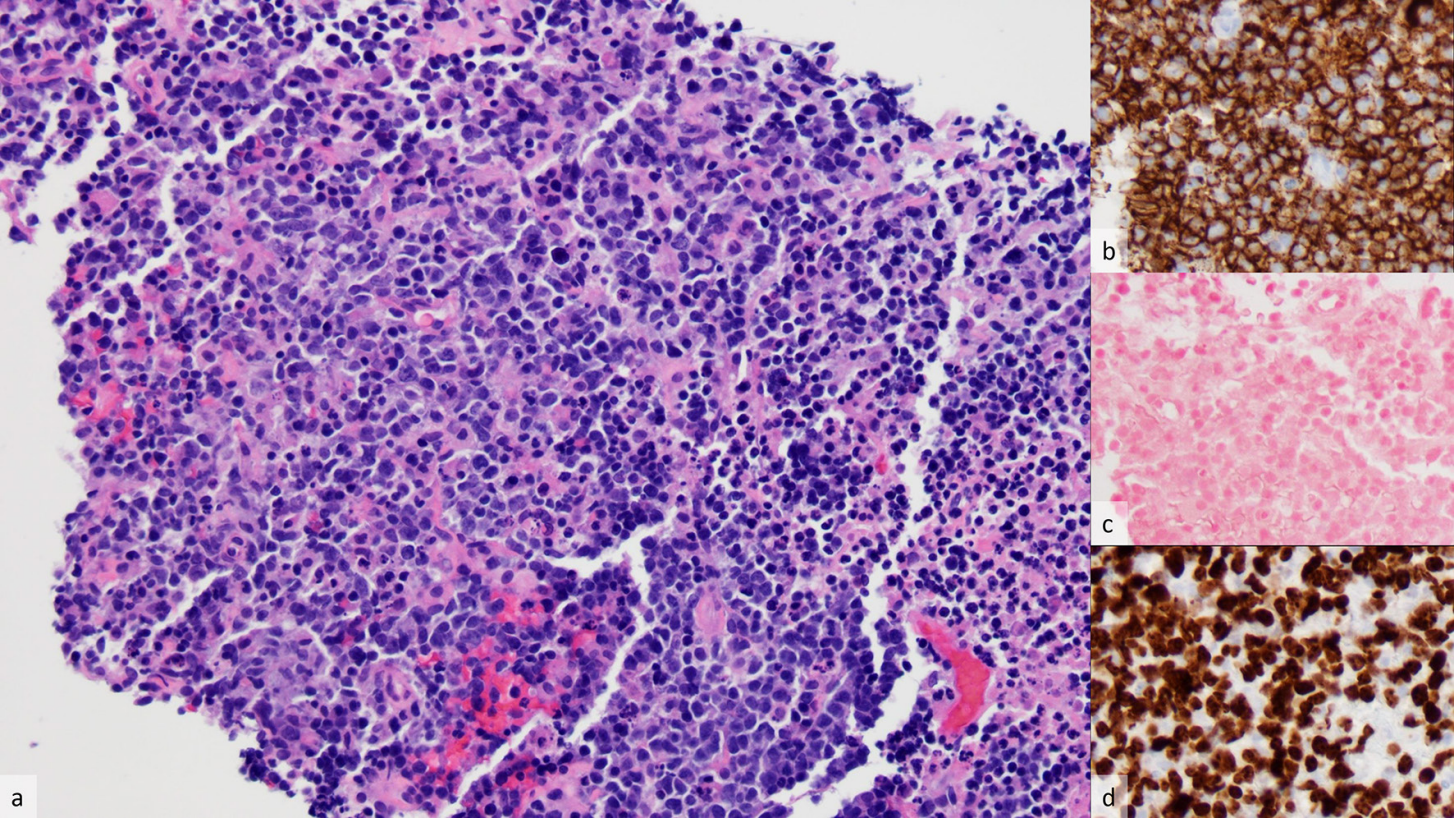
In (a) the needle core biopsy specimen demonstrates a diffuse proliferation of medium-to-large lymphoid cells manifesting enlarged nuclei with irregular borders and prominent nucleoli. Numerous mitotic figures and apoptotic bodies are present (hematoxylin and eosin, 200× magnification). (b) CD20 immunohistochemical stain shows B-cell differentiation of the neoplastic cells (200× magnification). (c) *In situ* hybridization for EBV reveals absence of reactivated EBV infection (200× magnification). (d) MIB-1 immunohistochemical stain is nearly 100% positive, consistent with the other aggressive findings in this case (200× magnification). EBV, epstein barr virus.

CT guidance was utilized in the biopsy of the large destructive sacral bone lesion. Pathological analysis revealed an EBV negative, diffuse large b-cell lymphoma with a high proliferation rate. Figure 6 confirming the diagnosis of PTLD. The additional thoracic findings were then presumed to be from PTLD given their rounded, mass-like appearance and marked FDG, and therefore biopsy of these lesions was not pursued.

## Discussion

PTLD, first described by Doak et al in 1968, covers a spectrum of lymphoproliferative disorders that can occur after solid organ or bone marrow transplantation.^1^ The incidence of PTLD is varied based on the type of organ transplantation, with intestinal and multiorgan transplants having the highest incidence of up to 20%. The incidence of PTLD following renal transplant, as in our case, is extremely low only occurring in 1–5% of post-transplant patients.^2–4^

WHO categorizations of PTLD are divided into early lesions, polymorphic PTLD, monomorphic PTLD, and classical Hodgkin lymphoma type PTLD. Characterization of PTLD is based on the histologic features, and biopsy is required to both establish the diagnosis and WHO category of PTLD. There is a bimodal distribution of incidence of PTLD in both age of onset, <10 years old and 60 years old, as well as in the time of disease onset following transplantation, with peak incidences at 1 year and 4–5 years post-transplant.^3,5^ Early lesions contain the reactive benign plasmacytic hyperplasias and present often within the first 12 months post-transplant.^6,7^ Polymorphic PTLD is the most common subtype can be either polyclonal or monoclonal and are intermediate grade. The monomorphic PTLD and classical Hodgkin lymphoma type categories includes high grade entities that are virtually identical to their respective lymphomas. These tend to occur many years after transplantation and include diffuse large B-Cell lymphomas, as in our case, as well as plasmacytoma-like and Hodgkin lymphoma-like monomorphic cell lines. The common pathogenesis involves chronic immunosuppression leading to reactivation of EBV from either the graft or the host tissues. This pathogenesis of EBV reactivation is seen more often in the reactive hyperplasia of PTLD that occur acutely following transplantation. In later stage malignant monomorphic disease, the aetiology is often unknown.

Localized disease is more common than multifocal organ involvement. The distribution of disease involvement, and rate of allograft involvement varies based on the type of allograft. Overall, nodal and abdominal involvement tend to be most common, with thoracic and neurologic manifestations seen less frequently.^8,9^ Musculoskeletal manifestations are exceedingly rare. In other reviewed case reports with osseous involvement, the histologic classifications reported were in the monomorphic category of PTLD. In a case with plasmacytoma cell myeloma subtype PTLD, imaging demonstrated a solitary expansile lytic osseous lesion, which later recurred elsewhere mimicking the imaging features of multiple myeloma.^10^ Another case report demonstrated a diffuse B-Cell lymphoma with a single mixed lytic and sclerotic lesion in the ilium, which was *T*_2_ hyperintense and enhanced with gadolinium on MRI.^11^ Our case is unique in that the patient presented with advanced and aggressive multifocal osseous disease mimicking the aggressive features of lytic metastasis. This case and the case described in Kushik et al draw a contrast in the MRI characteristics of musculoskeletal lesions with PTLD lesions found elsewhere in the body. Non-musculoskeletal lesions generally tend to be nonenhancing and intrinsically *T*_1_ and *T*_2_ hypointense, rather than the *T*_2_ hyperintense signal characteristics found in the osseous lesions.

Treatment of all non-localized PTLD involves reduction of immunosuppression. If remission is not achieved with immunosuppression reduction alone, or if the PTLD is EBV negative, concurrent chemotherapy with or without Rituximab is the next line of therapy.^8^ Treatment response is determined similarly to other lymphoproliferative and neoplastic processes, with CT and PET CT used to monitor lesion size and metabolic activity.

In this case, we illustrate the rare entity of primary aggressive bone involvement of PTLD in a patient 6 years after living donor renal transplant. While PTLD is a rare condition, the diagnosis should be considered in any patient presenting with multiple osseous lesions following solid organ or bone marrow transplant.

## Learning points

1. PTLD encompasses a spectrum of lymphoproliferative disorders that can occur after transplantation, ranging from benign hyperplasias to malignant lymphoma-like processes. PTLD can affect a variety of organ systems, however musculoskeletal manifestations are rare.
2. Imaging plays an integral role in the diagnosis and management of PTLD, as it is utilized in the initial diagnosis and staging of the disease, guiding biopsy of lesions, and determining treatment response.
3. Osseous manifestation can occur in the monomorphic category of PTLD and can mimic osseous metastases. PTLD should be considered in transplant recipients with multifocal osseous lesions.
